# Quality Assessment of Peripartum Care

**DOI:** 10.5812/ircmj.9069

**Published:** 2014-06-05

**Authors:** Farahnaz Changaee, Masoumeh Simbar, Alireza Irajpour, Soheyla Akbari

**Affiliations:** 1 Department of Nursing and Midwifery, Isfahan University of Medical Sciences, Isfahan, IR Iran; 2 Department of Midwifery and Reproductive Health, Shahid Beheshti University of Medical Sciences, Tehran, IR Iran; 3 Department of Medicine, Lorestan University of Medical Sciences, Khoramabad, IR Iran

**Keywords:** Peripartum, Health Care Quality, Iran

## Abstract

**Background:**

Maternal health improvement is one of the eight goals of the third millennium development, set in 2000. Pregnancy complications are the most important causes of maternal mortality worldwide. Proper and qualified health care access is one the most important factors for reducing maternal and neonatal mortality rates.

**Objectives:**

This study aimed to determine quality of peripartum care in Lorestan province in 2013.

**Materials and Methods:**

This was a descriptive cross-sectional study, in which quality of peripartum care was assessed among 200 women (sample size was determined according to other studies), referred to Lorestan province public hospitals. Quality assessment according to the WHO was used for the framework of structure, process and outcome. Data was collected by a researcher-made checklist, developed based on the administered instructions by Iran Health Ministry. The checklists were filled by observation. The calculated quality scores were expressed as percentage. SPSS version 18 was used for data analysis.

**Results:**

The mean percentages of compatibility with desirable situation were 54%, 57% and 66% in first, second and third stage of labor, respectively. The lowest scores were related to: Leopold maneuvers in the first stage, hand washing in the second stage and pulse control in the third stage of labor.

**Conclusions:**

Quality of peripartum care is moderate in Lorestan province, therefore, continuous evaluation of quality of care by administrators and hospital staff is essential to improve this quality and will ultimately result in maternal and neonatal health improvement.

## 1. Background

The ultimate goal of all efforts and human developments is health improvement. In this case, mothers and children as the vulnerable groups require more attention. Therefore prevention of maternal and neonatal death is one of the main pillars of social justice (1). Maternal health improvement is one of the eight goals of the third millennium development. Two important criteria to achieve this goal are reducing maternal mortality rate to three-quarters less than the rate in 1990 and women’s access to reproductive health services by 2015. Maternal mortality includes women’s death during pregnancy, child birth or within 42 days after child bearing (2). Maternal death is not only a health problem, but an indicator for community development and shows communities attitude towards maternal health (3). The most common causes of maternal mortality are complications of pregnancy that consist 18% of gynecological problems (4). Between 11% to 17% of maternal deaths occur during labor (5), of which more than 75% occur in two geographical regions; 53% in Africa and 25% in south-east Asia. Most of these deaths happen in developing countries, mainly in areas with lack of access to adequate health services. Average maternal mortality rate is 290 and 14 per 1000 live births in developing and developed countries, respectively (6). The reported maternal mortality rate in Iran was about 24.6 per 100000 live births in 2008 (7). According to national mortality reports, maternal mortality rate in Lorestan province is higher than the overall maternal mortality rate in Iran (8). The most prevalent cause of maternal mortality in Iran is bleeding. Different strategies like educating women, skilled attendant at delivery and proper care before and during pregnancy, during labor and postpartum are noticed as influencing factors to reduce maternal mortality (9). Annually near 50 million women suffer from maternal morbidity and maternal mortality related to pregnancy and delivery. Approximately, more than a quarter (300 million) of adult women in the developing countries are affected with pregnancy and delivery related injuries and illnesses (10).

Studies have shown that by providing quality and qualified health services and supplies, many of these deaths are preventable (6). Literature shows that labor under the guidance of educated and skilled care attendants will result in significant decrease in maternal mortality rate. Proper perinatal care is vital in reducing maternal mortality (11). Reduction in maternal and child mortality rates are among the main goals of WHO. WHO considers proper care during pregnancy, delivery and post-partum period as the most important strategies to achieve this goal (12). Despite the efforts of WHO, UNICEF, the World bank and other organizations for reducing maternal mortality rate by three quarters in 2015, there has been a little progress to achieve this goal and in some places the situation has even become worse (13). By providing quality care by skilled health care attendants, stillbirth rate has been reduced to 24%. To achieve desirable results, high quality care during pregnancy, labor and postpartum should be provided by educated and skilled health care providers (14). In Iran, near 98% of health care is provided by trained health care providers and antenatal health care coverage is 98%. Maternal and neonatal care, during labor is the most common cause of hospitalization (15), the goal of which is to achieve the highest level of maternal and neonatal health with minimal health interventions. Main labor care actions consist of procedures during first, second and third stages of labor (16). Health Ministry of Iran, considers reproductive health quality improvement as one of the priorities, according to the global goals of reproductive health strategies (3). As the reduction of maternal mortality is one of the most important goals of health ministry, the goal is set to be 18 per 100000 (this rate was 30 per 100000 in 1990 in Iran), but the reduction rate is slow (the reduction speed is 2.3% every year, while it should be 5.5%) (9). Therefore, fundamental actions should be performed to accelerate mortality rate reduction. Quality assessment and improvement are of the most important acts and the first steps to improve quality is to determine quality level. In a study entitled “quality of peripartum care offered to women admitted to delivery wards in selected hospitals of Kurdestan University of Medical Sciences” quality of care in first and third stages of labor were desirable while quality of care of second stage was intermediate (17). In our country, based on the third millennium development, maternal mortality rate has been reduced by about 50% and now is near 25 per 100000, which should reach 18 per 100000 by 2015 (9). Despite 98% antenatal care coverage in Iran, the maternal mortality rate is high and we are far from the goals of the third millennium (15). Therefore, quality of care assessment is essential. In Lorestan province of Iran, consistent with the goals of the health ministry, reduction in the maternal and neonatal mortality rates are among the main goals and assessment of quality of care is a the priority (18).

## 2. Objectives

This study aimed to assess quality of peripartum care, to show strengths and weaknesses as well as to define key interventional programs to improve quality of peripartum care in maternity hospitals affiliated to Lorestan University of Medical Sciences.

## 3. Materials and Methods

This descriptive cross sectional study was the quantitative part of a sequential quantitative-qualitative (explanatory) study. The second part examines the causes and strategies to improve the quality of care. The sample size for each group during labor stages, based on other studies, with a 95% confidence interval and d (7%), were approximately determined to be 200. Non-random quota sampling method was used to recruit 200 patients, among women referred to peripartum care unit of nine public hospitals affiliated to Lorestan University of Medical Sciences. Data collection was performed in nine public hospitals of Lorestan province Iran (cities: Khorramabad, Burujerd, Azna, Aligoodarz, Dorud, Kuhdasht, Aleshtar, Noorabad and Poldokhtar). Ten to 30 samples were selected to assess the provided care in different stages of labor (first, second and third stages of labor) in each hospital. Inclusion criteria were: age between 18-35 years, no previous medical problem, gestational age between 37-41 weeks, spontaneous onset of vaginal delivery, no contraindication for vaginal delivery, normal fetal heart rate and no complications in previous labor and delivery. Exclusion criteria were the occurrence of any of the above, due to which ten women were excluded and replaced.

A checklist was used to collect information about quality of peripartum care. Satisfaction questionnaires were completed by patients and checklists were completed by sampler observers. Peripartum care in different stages were observed and recorded. The checklist consisted of three sections: section one included 93 items to evaluate quality of care during the first stage of labor, like quality of communication between care-provider and the client, history-taking, vaginal examination, vital sign assessment and record, fetal heart assessment, assessment of uterus contractions and emotional support. Section two consisted of 43 items to evaluate quality of vital sign assessment, fetal heart rate assessment, hand washing, physical examination and emotional support. Section three included 71 items to evaluate quality of vital sign assessment, placenta examination, neonatal care and examination, episiotomy, emotional support and quality of records in the file. Items were rated as: completely performed (2 points), incompletely performed (1 point) or not performed (no points) (0). There was also a choice of not applicable, for items which may not be always required and were excluded from the overall calculation. CVI was used to assess the content validity of the method. Total scores for each area of care were calculated and expressed as percentages. Estimated percentage was considered as the percentage of compliance with desirable status. Scores considered not desirable, intermittent and desirable for scores were 0-33, 34-67 and 68-100 percent, respectively. To assess content validity, the checklist was evaluated by ten professors in the field of obstetrics and gynecology in Isfahan and Lorestan provinces and based on their comments, the checklist was revised. Concurrent reliability of the checklist was assessed and confirmed by Pearson correlation coefficient (0.86). To determine the validity of the questionnaire interviewing a limited number (15) of the target population was performed, to ask if the respondent did not understand the scientific and literary editing Reliability of satisfaction questionnaires was assessed using test retest, and the internal consistency was determined using Cronbach's alpha (0.81). Data were analyzed using SPSS software version 18.0 (SPSS Inc, Chicago, IL, USA). Data were presented as frequencies for categorical variables.

## 4. Results

Qualities of provided care to 200 pregnant women were assessed in the study. Most items of the first stage were considered desirable but some items like temperature, pulse control and performing Leopold maneuver were detected not to be desirable (Table 1). Most items of care procedures, during the second stage of labor, scored highly desirable but some items like blood pressure checking, pulse control and hand washing were detected not to be desirable (Table 2). Most items of care procedures in the third stage of the labor scored highly desirable but some, like blood pressure and pulse control and newborn assessment were considered not desirable (Table 3). Mean score of quality of care was intermittent in all three stages of labor (Table 4).

**Table 1.** Frequency of Provided Care to Women, With Different Qualities (Desirable, Intermittent and Not-Desirable) in the First Stage of Labor^a^

Care Procedure	Quality of Care			
	Desirable	Intermittent	Not Desirable	Mean Score
**Fetal heart rate assessment**	97	1.5	1.5	98
**History taking**	94.5	5.5	0	88.6
**Vaginal examination**	75.5	24.5	0	76.2
**Communication between care provider and client**	76	21	3	76
**Blood pressure control**	74	9	17	70
**Emotional support and physical care**	56	39	5	68.5
**Partograph use**	56.5	10	33.5	59
**Control of uterine contractions**	42	26.5	31.5	50
**Temperature control**	37.5	9.5	53	43
**Pulse control**	17	24	59	32.4
**Performing Leopold maneuver**	9.5	2	88.5	10.8

^a^ Data are presented as %.

**Table 2.** Frequency of Provided Care to Women, With Different Qualities (Desirable, Intermittent and Not-desirable) in the Second Stage of Labor^a^

Quality of Care	Care Procedure			
	Desirable	Intermittent	Not Desirable	Mean Score
**Episiotomy**	96	2	2	96.5
**Psychological support and physical care**	71	23	6	78
**Fetal heart Rate control**	75	22.5	2.5	77.3
**Assessment of delivery process**	84	13	3	77
**Assessment of delivery readiness**	29	63	18	53
**Blood pressure control**	36.7	11	52.7	41.5
**Pulse control**	15	18	67	27
**Hand washing**	11.5	16	72.5	25

^a^ Data are presented as %.

**Table 3.** Frequency of Provided Care to Women, With Different Qualities (Desirable, Intermittent and Not-Desirable) in the Third Stage of Labor^a^

Quality of care	Care Procedure			
	Desirable	Intermittent	Not Desirable	Mean Score
**Emotional support and physical care**	89	11	0	91
**Assessment of birth records**	89.4	10.6	0	90
**Assessment of placenta**	85.4	13.6	1	89.5
**Examination of episiotomy**	94	4	2	88
**Immediate care of the newborn**	71.5	22	6.5	74
**Blood pressure control**	47	12	41	52
**Newborn assessment**	33.5	20.5	46	50
**Pulse control**	14.6	22.1	63.3	31

^a^ Data are presented as %

**Table 4.** Frequency of Provided Care to Women, With Different Quality (Desirable, Intermittent and not-Desirable) in Three Stages of Labor^a^

Stages of Labor	Quality of Care			
	Desirable	Intermittent	Not Desirable	Mean Score
**First**	14	83	3	54.5
**Second**	24.6	71.4	4	57
**Third**	57.9	38.5	3.6	66

^a^ Data are presented as %.

## 5. Discussion

The results of current study showed that the quality of care provided in the first stage of labor in hospitals of Lorestan province were detected to be intermediate to desirable. In few care procedures like performing Leopold maneuver and pulse control the quality of provided care was not desirable. Quality of fetal heart rate assessment by Sonicaid in the first and second stage were scored higher than 90% that could be due to the easy nature of use and availability for replacing the assessment by Pinard. After fetal heart rate assessment by Sonicaid, history-taking scored higher than other care procedures. Our results showed that quality of performing Leopold maneuver is not desirable. Leopold maneuver is an easy way to determine fetal position, presentation and descent. In addition, fetus weight could be estimated by this maneuver, all showing that this maneuver is an important and useful method that could be performed in a short time. One of the explanations for this finding may be failure to understand the importance of this maneuver by care providers (19). It could be also be due to inadequacy of the midwifery personnel. The standard rate for midwifery personnel presence for pregnant women in labor is one for one (20) but this is not possible in Lorestan province. Therefore, lack of midwives results in pressure overload and insufficient efficacy of midwifery care. Assessment of vital signs was not desirable in our study. Vital sign control, like pulse control in the first stage should be performed every 4 hours (21) and it is one of the crucial acts during labor In this study the quality of care using partograph in Lorestan province was intermediate. Nowadays usage of partograph causes improvement of quality of maternal and neonatal care and immediate identification of any problems. This graph shows the details of labor to midwife visually (22) and when electronic fetal monitoring is impossible, this can provide precise evaluation of maternal and neonatal health and a better assessment of health attendants by administrators (23). In evaluation of the second stage, quality of hand washing and pulse control scored less that 33% and had not a desirable quality. WHO has mentioned hand washing as one of the five elements of patient safety and it has also been considered as the first and the most important need in labor management. Hand washing is more important than antibiotic administration in controlling infections during different stages of the labor (24). In Lorestan province the quality of this service is not desirable and one of the explanations for this finding is its underestimation by health attendants. Educational courses on this subject may be helpful. Physical care and emotional support in all stages had been scored desirable. The advantages of these acts pain and anxiety reduction and increasing the rate of spontaneous labor (25). One of the important issues in this area is continuous care by midwife, the core component of the emotional support. Continuous midwifery care will help the maternal body to produce endorphins (26). In a study conducted by Truijens et al. it was demonstrated that existence of an accompanying person for each mother during labor will result in reduction of oxytocin consumption and labor complications (27). In our hospitals, it is not possible for fathers to company their spouses during labor, while their accompanying will be helpful during labor. In Lorestan province midwives pay attention to this care and provide this care with desirable quality.

Fetal heart rate assessment should be performed every 15 minutes in low risk deliveries and every five minutes in high risk deliveries (28). The results of this study confirmed that in Lorestan province this procedure is being performed based on WHO recommendation. Preparing the mother for the second stage of the labor and teaching her to cooperate in the second stage is one of the most important steps in advancements of the second stage of labor (17). Our results showed that this step is also provided with high quality in Lorestan province. Performing episiotomy is another care procedure, provided with high quality in Lorestan province. Nowadays the trend for performing episiotomy has reduced due to the complications like flatus and fecal incontinency. Episiotomy should be chosen in cases withe fetal indications, like shoulder dystocia, breech delivery exists and in cases in which forceps or vacuum usage are indicated (29). It seems peripartum care providers need training courses, being familiar with recent researches and guidelines about that. In the third stage, some care procedures like neonatal examination and pulse control had an average quality and their total score was less than 67%. Neonatal examination is important because it makes early diagnosis of fetal abnormalities possible (30) and therefore it should be improved. Blood pressure measurement during labor is important for evaluating maternal health and diagnosis of critical disorders like bleeding (31). Based on numerous studies, bleeding is one of the major causes of maternal death due to pregnancy and child birth, worldwide, preventable by early detection and treatment (32). Hypotension is a sign of bleeding that can prevented by precise control. Due to results of this study, the quality of this care was intermediate in Lorestan province and needs improvement. According to the results of current study, quality of peripartum care in most areas, especially in critical areas of care were considered desirable or intermediate and in few areas the quality of care was not desirable. The weakness was mostly due to replacement of old devices medical with new ones. In this study, we applied the guidelines of ministry of health about various stages of peripartum care for quality assessment. According to the results, holding training courses for the care providers, developing peripartum care protocols, promotion of partograph usage, monitoring peripartum care providers practices, including simple cares like pulse control and hand washing, improved communication with parturient, familiarizing patients with labor and childbirth environment and obtaining their satisfaction are recommended. Limitation of this study is that its findings are applicable for all communities, but with broad researches in different cities this problem can be solved.

## References

[1] Simbar M, Dibazari ZA, Saeidi JA, Majd HA (2005). Assessment of quality of care in postpartum wards of Shaheed Beheshti Medical Science University hospitals, 2004.. Int J Health Care Qual Assur Inc Leadersh Health Serv..

[2] World Health Organization UNICEF UNFPA and The World Bank.. (2010). Trends in maternal mortality: 1990 to 2008 Estimates developed by WHO, UNICEF, UNFPA and The World Bank.:.

[3] Ministry of Health and Medical Education. (2011.). [Health department Maternity care plan]..

[4] Mutihir JT, Utoo BT (2011). Postpartum maternal morbidity in Jos, North-Central Nigeria.. Niger J Clin Pract..

[5] Torkzahrani S, Khazaiyan S, Shaykhan Z, Akbarzadeh A (2010). Satisfaction of mothers with the performance of health care providers in postpartum wards of hospitals in Zahedan.. J Nurs Midwifery Shahid Beheshti Univ Med Sci Health Serv..

[6] World Health Organization UNICEF World Bank United Nations Population Fund.. (2007). Maternal mortality in 2005 : estimates developed by WHO, UNICEF, UNFPA, and The World Bank..

[7] United Nations Children’s Fund. (2007). Progress for children A world fit for children statistical review..

[8] World Health Organization. (2009). Regional office for the Eastern Mediterranean Making Pregnancy Safer Data Base in the EMR.:.

[9] Ministry of Health and Medical Education. (2011.). Lorestan University of Medical Sciences and Health Department reported.

[10] World Health Organization. (2005.). Strategic direction for accelerating the reduction of Maternal Mortality in EMRO Regional office for the Eastern Mediterranean..

[11] Liang J, Li X, Dai L, Zeng W, Li Q, Li M (2012). The changes in maternal mortality in 1000 counties in mid-Western China by a government-initiated intervention.. PLoS One..

[12] Van Hoof TJ, Casey BA, Tate JP, Linnane JJ, Petrillo MK, Meehan TP (2000). The status of prenatal care among Medicaid Managed Care patients in Connecticut.. Eval Health Prof..

[13] Kidney E, Winter HR, Khan KS, Gulmezoglu AM, Meads CA, Deeks JJ (2009). Systematic review of effect of community-level interventions to reduce maternal mortality.. BMC Pregnancy Childbirth..

[14] Yakoob MY, Ali MA, Ali MU, Imdad A, Lawn JE, Van Den Broek N (2011). The effect of providing skilled birth attendance and emergency obstetric care in preventing stillbirths.. BMC Public Health..

[15] Ministry of Health and Medical Education. (2011.). According to Health Minister Mama Day Persian date Ordibehesht 1390.

[16] World health organization. (1996). Maternal health and safe motherhood programme Division of family health Mother baby package..

[17] Simbar M, Ahmadi M, Ahmadi G, Majd HR (2006). Quality assessment of family planning services in urban health centers of Shahid Beheshti Medical Science University, 2004.. Int J Health Care Qual Assur Inc Leadersh Health Serv..

[18] World Health Organization.. (2010.). Maternal mortality..

[19] Toosy Najafabadi Z (1999). An evaluate of prenatal parturient and postpartum care and related effective factors on it in Tooiserkan City Master of Health Services Management thesis Health College and Research Institute MS Thesis..

[20] Simbar M, Ghafari F, Zahrani ST, Majd HA (2009). Assessment of quality of midwifery care in labour and delivery wards of selected Kordestan Medical Science University hospitals.. Int J Health Care Qual Assur..

[21] Azari S, Sehhati F, Ibrahimi H (2010). The quality of care in public and private hospitals after cesarean delivery in Tabriz .. The first International Conference on Health Promotion and Education..

[22] Omidvar A, Ja'farnejad F, Noorani S, Esmaeili H (2003). A comparative study of physical and psychosocial care in primiparous and multiparous women in Mashad University hospitals.. J Nurs Midwifery..

[23] Lorestan University of Medical Sciences. (2008.). Deputy of Research and Technology Research Priorities Priorities of Midwifery..

[24] World Health Organization.. (2010). Global Health Workforce Health Alliance Workers for all and all for health workers Media centre Aliance News Digest.:.

[25] Ministry of Health Medical Education. (2009.). Office of Population and Family Health A guide to natural childbirth and provide pharmacologic and nonpharmacologic labor.

[26] Barbosa da Silva FM, Rego da Paixao TC, de Oliveira SM, Leite JS, Riesco ML, Osava RH (2013). [Care in a birth center according to the recommendations of the World Health Organization].. Rev Esc Enferm USP..

[27] Truijens SE, Pommer AM, van Runnard Heimel PJ, Verhoeven CJ, Oei SG, Pop VJ (2014). Development of the Pregnancy and Childbirth Questionnaire (PCQ): evaluating quality of care as perceived by women who recently gave birth.. Eur J Obstet Gynecol Reprod Biol..

[28] World Health Organization. (2006.). Clean care is safer care..

[29] World Health Organization Department of Reproductive Health and Research United Nations Population Fund UNICEF World Bank. (2003). Managing complications in pregnancy and childbirth: a guide for midwives and doctors..

[30] United Nations Population Fund.. (2009). State of World population Maternal Health Quality of Maternal Health.:.

[31] Bhattacharyya S, Srivastava A, Avan BI (2013). Delivery should happen soon and my pain will be reduced: understanding women's perception of good delivery care in India.. Glob Health Action..

[32] Knape N, Schnepp W, Krahl A, Zu Sayn-Wittgenstein F (2013). [The efficiency of one-to-one support during labour - a literature analysis].. Z Geburtshilfe Neonatol..

